# Supplementary material for: Biclustering reveals potential knee OA phenotypes in exploratory analyses: Data from the Osteoarthritis Initiative
Source: PLoS One. 2022 May 24;17(5):e0266964. doi: 10.1371/journal.pone.0266964 (PMC9129051; doi:10.1371/journal.pone.0266964)
Supplement: S1 Table — (PDF) [file pone.0266964.s001.pdf]

S1 Table. OAI Variable descriptions and key to NDA dataset

Description of the variable	OAI variable used	NDA variable used
Female sex	P02SEX	gender
Hispanic or Latino, self-reported	P02HISP	ethnicity
White or Caucasian race	P02RACE	race
Black or African American race	P02RACE	race
Asian race	P02RACE	race
SF-12 Physical Health Summary	V00HSPSS	physical
SF-12 Mental Health Summary	V00HSMSS	mental
Marital status: Widowed	V00MARITST	curr_marital_st
Marital status: Divorced	V00MARITST	curr_marital_st
Marital status: Separated	V00MARITST	curr_marital_st
Marital status: Never married	V00MARITST	curr_marital_st
Doing unpaid work for family business	V00CEMPLOY	cemploy
Not working in part due to health	V00CEMPLOY	cemploy
Not working other reasons	V00CEMPLOY	cemploy
Currently employed	V00CUREMP	curemp
Yearly income >50K (calc)	V00INCOME2	income2
Have medical insurance	V00MEDINS	medins
Age (calc, used for study eligibility)	V00AGE	ageyears
Highest grade or year of school completed (calc)	V00EDCV	edcv
How many alcoholic drinks in typical week, past 12 months	V00DRNKAMT	drnkamt
Comorbidity Score	V00COMORB	comorb
Center for Epidemiologic Studies Depression Scale (CES-D) Score (calc)	V00CESD	cesd_score
Physical Activity Scale for the Elderly	V00PASE	pase
Blood pressure: systolic (mm Hg)	V00BPSYS	bpsys
Blood pressure: diastolic (mm Hg)	V00BPDIAS	bpdias
Body mass index (calc)	P01BMI	bmi
Fallen and landed on floor or ground, past 12 months	V00FALL	fall
Smoked at least 100 cigarettes (5 packs) in entire life	V00SMOKE	smoke
In the past, did drink more beer, wine or liquor than now	V00DRKMORE	drkmore
Total number of medications recorded	V00RX30NUM, V00RX30	rx30num, rx30
Bring in or identify ALL prescription medications taken, past 30 days	V00RX30	rx30
Bring in or identify SOME prescription medications taken, past 30 days	V00RX30	rx30
Did not take any medications, past 30 days	V00RX30	rx30
Used analgesic in last 30 days	V00RXANALG	rxanalg
Used narcotic analgesic in last 30 days	V00RXNARC	rxnarc
Injected corticosteroid in last 30 days	V00RXISTRD	rxistrd
Used COX-II inhibitor in last 30 days	V00RXCOX2	rxcox2
Used NSAID in last 30 days	V00RXNSAID	rxnsaid
Ever had hip replacement surgery where all or part of joint was replaced	P01HRS	hrs
Doctor ever said participant broke or fractured bone after age 45	V00BONEFX	bonefx
Doctor ever said participant fractured spine or vertebrae	V00SPNFX	spnfx
Repeated chair stand: pace in stands/sec (calc)	V00CSPACE	cspase
Single chair stand	V00CSTSGL	cstsgl
Able to complete 5 repeated chair stands	V00CS5	cs5
20-meter walk: pace (m/sec) (calc)	V0020MPACE	w20mpace
400 meter walk	V00400MTIM	w400mtim
Family history of TKR	P02FAMHXKR	famhkkr
Presence of hip pain (right), aching or stiffness: any, past 12 months (includes pain in groin and in front and sides of upper thigh)	P01HPNR12_1: Yes	hpnr12
Presence hip pain (left), aching or stiffness: any, past 12 months (includes pain in groin and in front and sides of upper thigh)	P01HPNL12_1: Yes	hpnl12
Presence of back pain (any), past 30 days	P01BP30	bp30
Presence of right hip pain, aching or stiffness: more than half the days of a month, past 12 months (calc)	P01HPR12CV	hpr12cv
Presence of left hip pain, aching or stiffness: more than half the days of a month, past 12 months (calc)	P01HPL12CV	hpl12cv
**Ever injured badly enough to limit ability to walk for at least two days**	P01INJL, P01INJR	injl, injr
**Ever have knee surgery or arthroscopy**	P01KSURGL, P01KSURGR	ksurgl, ksurgr
**Ever have knee arthroscopy (where they put a scope in knee)**	P01ARTL, P01ARTR, P01KSURGL, P01KSURGR	artl, artr, ksurgl, ksurgr
**At least one arthroscopy to repair knee injury**	P01ARTLINJ, P01ARTRINJ, P01KSURGL, P01KSURGR	artlinj, artrinj, ksurgl, ksurgr
**Ever have meniscectomy (where they repaired or cut away torn meniscus or cartilage)**	P01MENL, P01MENR, P01KSURGL, P01KSURGR	menl, menr, ksurgl, ksurgr
**At least one meniscectomy to repair an injury**	P01MENLINJ, P01MENRINJ, P01KSURGL, P01KSURGR	menlinj, menrinj, ksurgl, ksurgr
**Ever have ligament repair surgery**	P01LRL, P01LRR, P01KSURGL, P01KSURGR	lrl, lrr, ksurgl, ksurgr
**WOMAC Pain Score (calc)**	V00WOMKPL, V00WOMKPR	womkpl, womkpr
**KOOS Pain Score**	V00KOOSKPL, V00KOOSKPR	kooskpl, kooskpr
**WOMAC Stiffness Score (calc)**	V00WOMSTFL, V00WOMSTFR	womstfl, womstfr
**KOOS Symptoms Score (right/**	V00KOOSYML, V00KOOSYM	koosyml,koosymr
**WOMAC Disability Score (calc)**	V00WOMADLL, V00WOMADLR	womadll, womadlr
**Isometric strength: left leg weight (N)**	V00LWGTL, V00LWGTR	rlwgt, llwgt
**Isometric strength: left knee extension, severity of pain (calc)**	V00LEXP1CV, V00REXP1CV	lexp1cv, rexp1cv
**Isometric strength: right knee flexion, severity of pain (calc)**	V00LFXP1CV, V00RFXP1CV	lfxp1cv, rfxp1cv
**Right knee exam: flexion contracture/hyperextension, degrees (contracture positive) (calc)**	V00LKFHDEG, V00RKFHDEG	rkfhdeg, lkfhdeg
**knee exam: alignment, degrees (valgus negative) (calc)**	V00LKALNMT, V00RKALNMT	lkalnmt, rkalnmt
**Flexion MAX Force**	V00rfmaxf, V00lfmaxf	rfmaxf, lfmaxf
**Flexion Speed of Force Relaxation**	V00lfSFR, V00rfSFR	lfsfr, rfsfr
**Extension Speed of Force Production**	V00leSFP, V00reSFP	resfp, lesfp
**Extension Speed of Force Relaxation**	V00leSFR, V00reSFR	resfr, lesfr
**Flexion Speed of Force Production**	V00lfSFP, V00rfSFP	lfsfp, rfsfp
**Knee pain, aching or stiffness: any, past 12 months**	P01KPNL12, P01KPNR12	kpnl12, pnr12
**Knee exam: presence of patellar quadriceps tendinitis, pain/tenderness at any four sites**	V00LKPATPN, V00RKPATPN	lkpatpn, rkpatpn
**Knee exam: effusion, bulge sign positive**	V00LKEFFB, V00RKEFFB	lkeffb, rkeffb
**Knee exam: effusion, patellar tap positive**	V00LKEFFPT, V00RKEFFPT	rkeffpt, lkeffpt
**Knee is to tender to examine**	V00LKEFFPT, V00RKEFFPT	rkeffpt, lkeffpt
**presence of knee flexion pain/tenderness on knee exam**	V00RKRFXPN, V00LKRFXPN	lkrfxpn, rkrfxpn
**Presence of lateral tibiofemoral pain/tenderness on knee exam**	V00LKLTTPN, V00RKLTTPN	lklttpn, rklttpn
**Presence of medial tibiofemoral pain/tenderness on knee exam**	V00LKMTTPN, V00RKMTTPN	lkmttpn, rkmttpn
**Presence of anserine bursa, pain/tenderness on exam**	V00LKABPN, V00RKABPN	lkabpn, rkabpn
**Knee exam: patello-femoral crepitus present on exam**	V00LKPFCRE, V00RKPFCRE	lkpfcre, rkpfcre
**Knee exam: patellar grind, painful or tender behind knee cap**	V00LKPGDPN, V00RKPGDPN	lkpgdpn, rkpgdpn
**BL knee symptom status**	P01LKSX, P01RKSX	lksx, rksx

Bold: knee level variable
