# Supplementary material for: Biclustering reveals potential knee OA phenotypes in exploratory analyses: Data from the Osteoarthritis Initiative
Source: PLoS One. 2022 May 24;17(5):e0266964. doi: 10.1371/journal.pone.0266964 (PMC9129051; doi:10.1371/journal.pone.0266964)

S1 Figures.

Spaghetti plots for the WOMAC pain score trajectory groups over time (t), showing how knees express the basic patterns (stable, up, down, irregular/erratic) with error, for: Group 1, Improvement trajectory (A); Group 2, Stable trajectory (B); Group 3, Worsening trajectory (C); Group 4, Irregular trajectory (D).

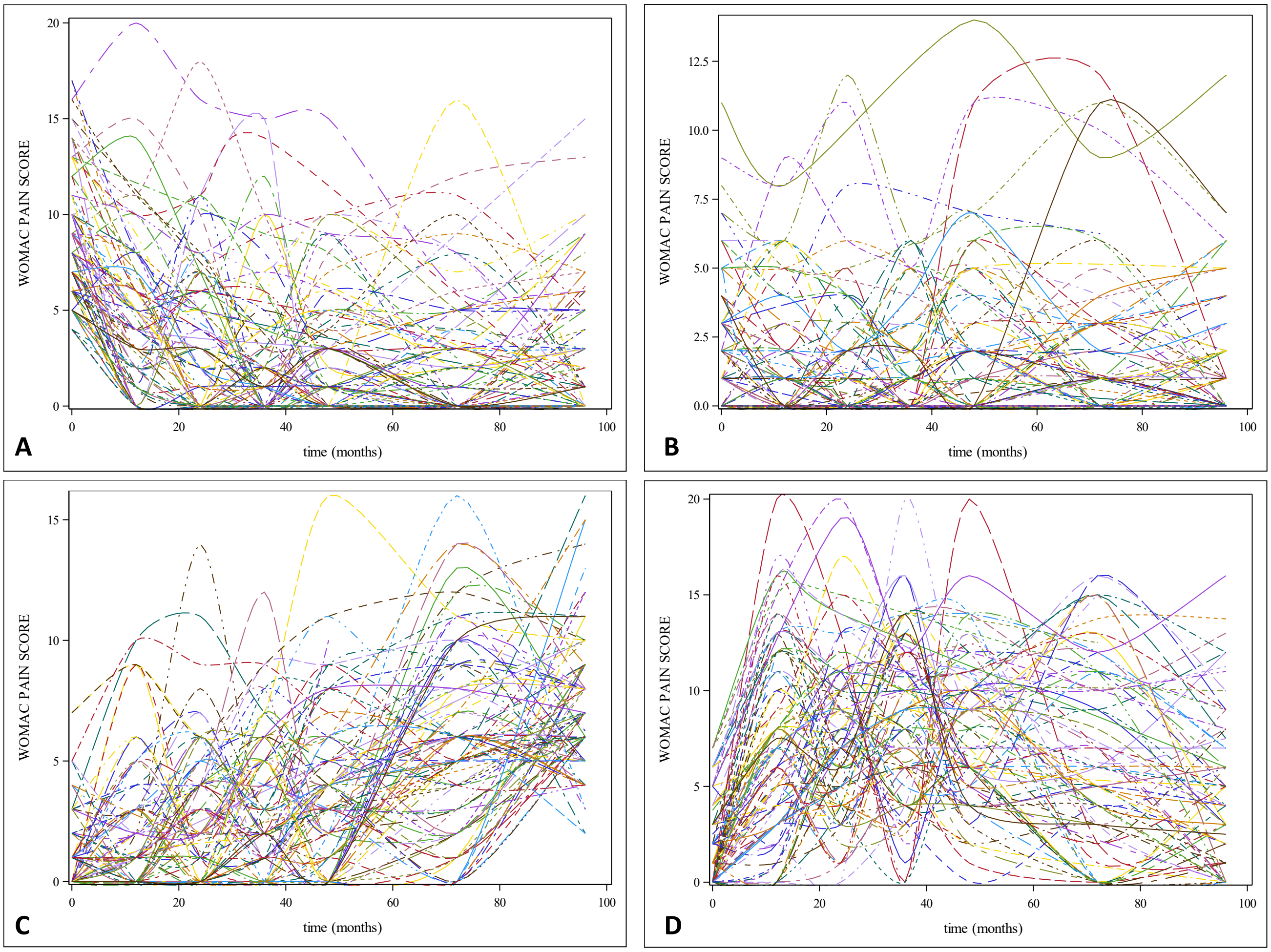

Supplement: S1 Fig — (PDF) [file pone.0266964.s002.pdf]
